# Nasal polyp syndrome: a patient-centred term for CRSwNP by EUFOREA

**DOI:** 10.3389/falgy.2024.1372919

**Published:** 2024-03-20

**Authors:** T. Teeling, C. Djouder, H. Laurens, J. H. Preyra, C. M. E. Shire, E. Van Staeyen, D. M. Conti, G. K. Scadding, P. W. Hellings

**Affiliations:** ^1^ Patient Advisory Board of the European Forum for Research and Education in Allergy and Airway Diseases, Brussels, Belgium; ^2^ The European Forum for Research and Education in Allergy and Airway Diseases Scientific Expert Team Members, Brussels, Belgium; ^3^ Escuela de Doctorado UAM, Centro de Estudios de Posgrado, Universidad Autónoma de Madrid, Calle Francisco Tomás y Valiente, n° 2. Ciudad Universitaria de Cantoblanco, Madrid, Spain; ^4^ Department of Allergy & Rhinology, Royal National ENT Hospital, London, United Kingdom; ^5^ Division of Immunity and Infection, University College, London, United Kingdom; ^6^ Allergy and Clinical Immunology Research Unit, Department of Microbiology and Immunology, KU Leuven, Leuven, Belgium; ^7^ Clinical Department of Otorhinolaryngology, Head and Neck Surgery, University Hospitals Leuven, Leuven, Belgium; ^8^ Department of Otorhinolaryngology, Laboratory of Upper Airways Research, University of Ghent, Ghent, Belgium

**Keywords:** EUFOREA, CRSwNP, Nasal Polyp Syndrome, global CRSwNP awareness day, patient-provider communications, lay language, patient advisory board

## Abstract

Chronic Rhinosinusitis with Nasal Polyps (CRSwNP) is a chronic inflammatory disease of the nose and paranasal sinus cavities that significantly affects well-being and social function, particularly in young adults and middle-aged populations. CRSwNP is a common health condition in the Western world, with an estimated prevalence of 3%. Despite worldwide evidence-based treatment guidelines such as the European Position Paper on Rhinosinusitis and Nasal Polyps (EPOS) 2020 and the European Forum for Research and Education in Allergy and Airway Diseases (EUFOREA) chronic rhinosinusitis (CRS) pocket guide, a significant number of patients remain undiagnosed and/or uncontrolled with repeated oral corticosteroids (OCS) treatments and/or (multiple) endoscopic sinus surgeries (ESS).

## Introduction

Chronic Rhinosinusitis with Nasal Polyps (CRSwNP) is a chronic inflammatory disease of the nose and paranasal sinus cavities that significantly affects well-being and social function, particularly in young adults and middle-aged populations (1). CRSwNP is a common health condition in the Western world, with an estimated prevalence of 3% (1–3). Despite worldwide evidence-based treatment guidelines such as the European Position Paper on Rhinosinusitis and Nasal Polyps (EPOS) 2020 (4) and the European Forum for Research and Education in Allergy and Airway Diseases (EUFOREA) chronic rhinosinusitis (CRS) pocket guide (5), a significant number of patients remain undiagnosed and/or uncontrolled with repeated oral corticosteroids (OCS) treatments and/or (multiple) endoscopic sinus surgeries (ESS).

Patients with CRSwNP exhibit sinonasal symptoms like nasal obstruction, smell dysfunction with anosmia in a significant proportion, persistent nasal discharge, and/or facial pain (1). Apart from sinonasal symptoms, CRSwNP is associated with an increased risk of asthma, otitis media, depression and social dysfunction (6). Similarly important is the extensively studied association between CRSwNP and sleep disturbance, which is linked to cardiovascular and cerebrovascular disease and reduced quality of life for both the patient and their partner (7–9). The impact of CRSwNP on quality of life (QoL) has been observed to be equivalent to other chronic conditions such as chronic obstructive pulmonary disease (COPD), congestive heart failure, and diabetes (10, 11). Yet in comparison to asthma, respiratory allergies and atopic dermatitis (12–14), few global patient initiatives have been undertaken to bring the physical and psychosocial burden of disease of CRSwNP to the attention of health policymakers, the general public, or physicians. Although it is commonly acknowledged that upper and lower airway diseases are linked to inflammation in the airways (15), few global initiatives have been undertaken to emphasise the patient perspective and the impact of diseases in both the upper and lower airways (16–18).

The substantial burden of CRSwNP on both the patient as well as the society warrant the implementation of improved treatment options and the reorganisation of existing care pathways (19, 20). The substantial financial burden associated with CRSwNP arises from expenditures associated with healthcare use, absenteeism from work and school, and decreased job productivity (21, 22).

**Figure 1:**
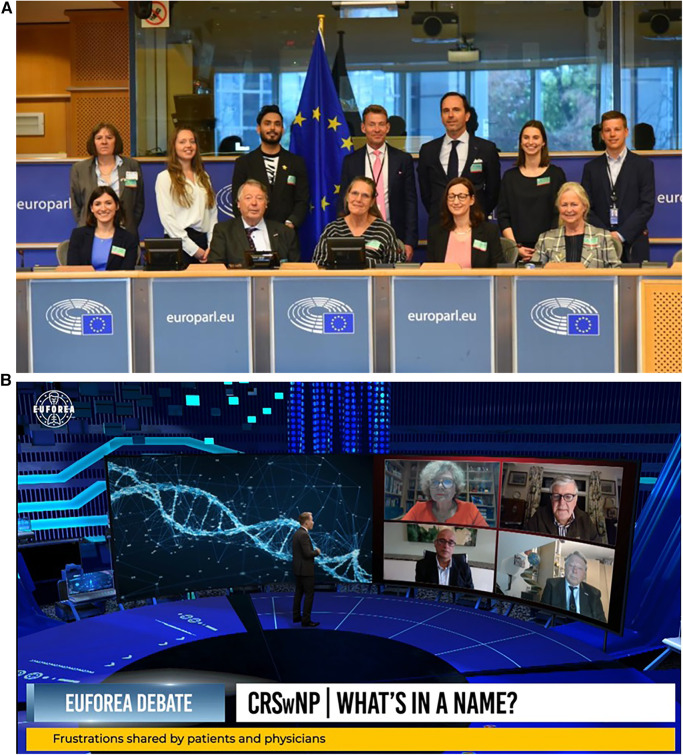
(**A**) Group picture of debate at the first annual Global Chronic Rhinosinusitis with Nasal Polyps (CRSwNP) awareness day 2022. (**B**) EUFOREA Innovation Forum debate on the need for a lay language term to improve patient-provider communications.

During the EUFOREA's first annual Global Chronic Rhinosinusitis with Nasal Polyps Awareness Day on April 20, 2022 in the European Parliament in Brussels (Figure 1A) a moderator and a panel consisting of global experts in CRSwNP and patient representatives from EUFOREA's patient advisory board (PAB) took part in an informal debate on the burden of CRSwNP (Figure 1B) on the nature of the complex names “CRSwNP” or “Chronic Rhinosinusitis with Nasal Polyps’ for patients, for health policy makers and for physicians. Discussions among members of EUFOREA's patient advisory board revealed the challenges faced by patients with CRSwNP as they not only navigate conversations with family, friends and peers about the burden of their disease and its associated comorbidities, but also as they seek out reliable information about their disease online to play an active and empowered role in as they discuss treatment options with their healthcare team. The difficult and inconsistent naming used for CRSwNP by patients and healthcare professionals alike not only exacerbates patients” reported perception of a lack of resources about their disease, but also hinders patients’ involvement in shared decision making with their healthcare team.

The definition of CRSwNP was proposed at the first EPOS expert committee meeting in Amsterdam in 2001. This term encompasses multiple facets of the disease, including its inflammatory nature, the involvement of the nasal and paranasal sinus cavities, as well as the presence of bilateral sino-nasal polyps found either during nasal endoscopy or on CT scans. The name CRSwNP also allows the phenotypic distinction of CRSwNP from those patients without the presence of nasal polyps, defined as CRSsNP with “s” standing for “sin” or without. In addition, the term CRSwNP also avoids confusion with a condition called “adenoid hypertrophy”, which is often referred to as “nasal polyps” in lay language. Since the release of the first EPOS consensus document, experts worldwide have adopted the term CRSwNP in all guidelines and publications given the best coverage of the disease with phenotypic definition of the presence of nasal polyps.

In spite of all benefits and accuracy of the name CRSwNP, the introduction of a lay language term for CRSwNP is considered useful by patients in the light of the following considerations:

- CRSwNP is barely pronounceable by patients and even more so by non-native English-speaking patients and even physicians, hence leading to several uncomfortable situations where patients can neither express nor discuss their disease with physicians and peers as the name is too complex, and
- CRSwNP is still not a well-known disease entity beyond the ear, nose and throat (ENT) specialist community, and
- CRSwNP does not cover the complexity of the disease with most severe patients having co-morbidities like middle ear problems, lower airways disease (including asthma), aspirin intolerance and/or allergies, and
- CRSwNP disease often requires a multispeciality approach for reaching optimal disease control.

This exercise in patient advocacy has been done in the past and the literature shows how terminology has been divided when naming diseases. As a result, there are terms used by the medical community and others used by the patient community that refer to the same disease or event and are equally accepted. Examples included in Table 1:

**Table 1:** Examples of lay language terms vs. medical terms. 
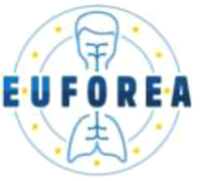

Lay language terms	Medical terms
Hay fever	Allergic rhinitis
Eczema	Atopic dermatitis
Hearth attack	Myocardial infarction
Asthma attack	Asthma exacerbation
Stroke	Cerebrovascular accident
High cholesterol	Hypercholesterolemia
Vomiting	Emesis
Itching	Pruritus
Fainting	Syncope
Nose bleeds	Epistaxis

**Figure 2:**
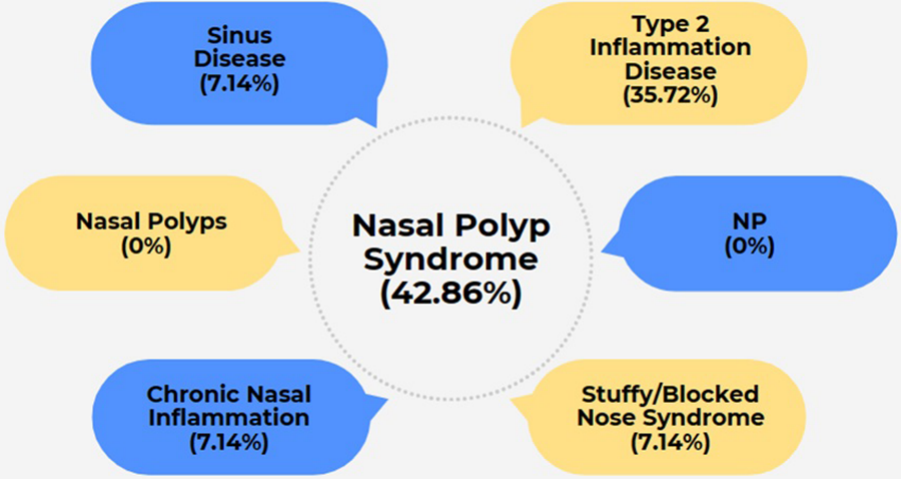
Lay language terms proposed by the EUFOREA patient advisory board along with poll results for preferred term.

**Figure 3:**
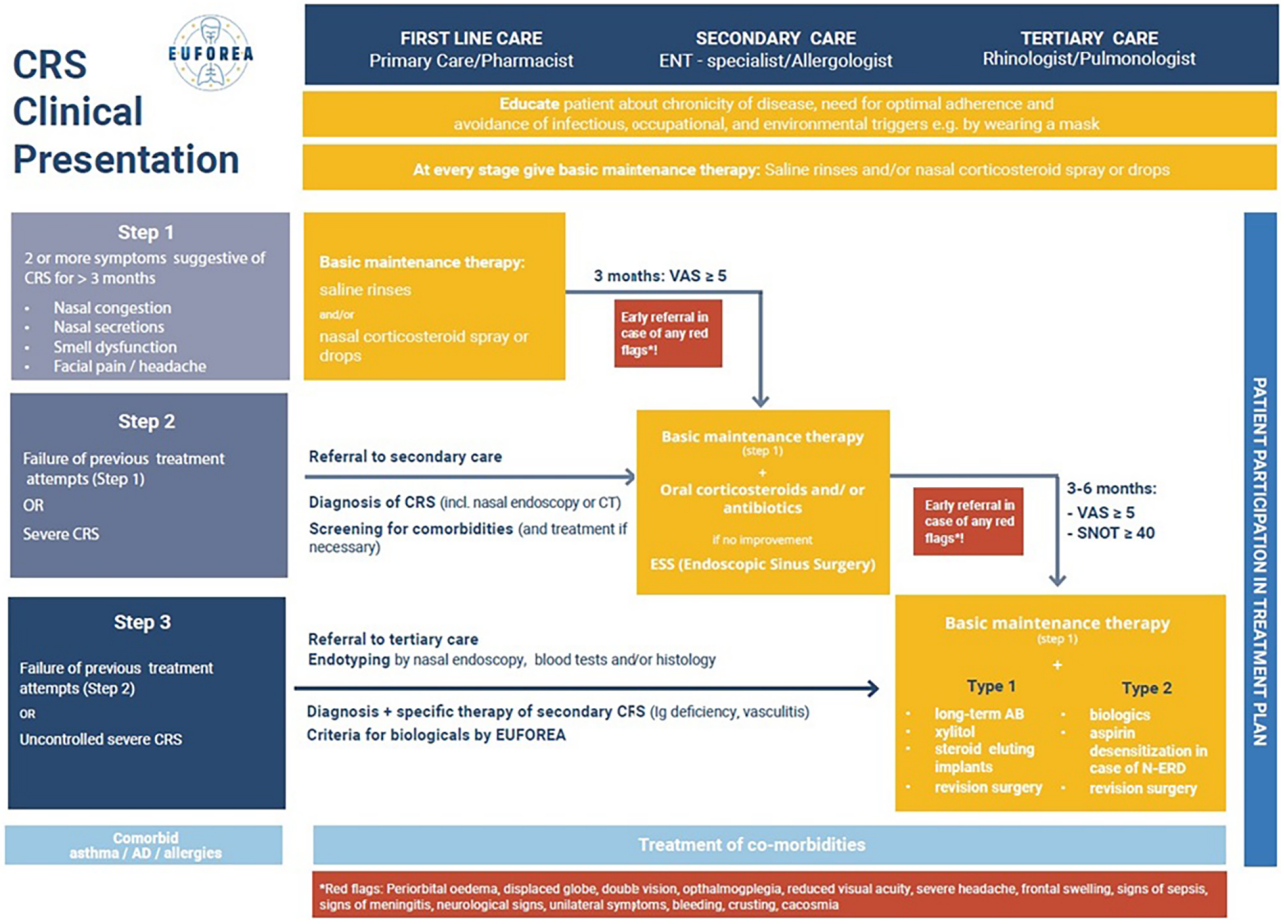
EUFOREA chronic rhinosinusitis treatment algorithm.

Following the PAB meeting of EUFOREA in September 2022, European patients suffering from long-standing severe CRSwNP expressed the suggestion to implement a new lay language term for CRSwNP (16–18), with the ambition to find a consensus amongst patients on how to properly name their condition in a simple, comprehensive and appealing way for all stakeholders. The following names for CRSwNP were discussed during two virtual meetings with a panel of over 50 European patients as well as over 30 experts affiliated with the EUFOREA expert panel teams in 2022 and 2023: Nasal Polyp Syndrome (NPS), Sinus Disease, Type 2 Inflammation Disease, Nasal Polyps, NP, Chronic Nasal Inflammation, or Stuffy/Blocked Nose Syndrome. After 2 rounds of discussions with patients and exploration with expert colleagues, in which some of the names were dropped and others proposed, the majority of patients have opted for the name Nasal Polyp Syndrome (Figure 2). This name was also approved by experts involved in the recent EUFOREA new pocket guide and treatment algorithm for chronic rhinosinusitis (Figure 3).

The newly proposed name “Nasal Polyp Syndrome” by EUFOREA is endorsed by the CRS expert panel of EUFOREA and the EUFOREA patient advisory board members as it carries the following advantages:

- Simplicity in pronunciation
- Simplicity in communication about the disease with the patient, with physicians and with health policy makers
- Shorter name than CRSwNP, both in full as well as in abbreviated form
- Covering the burden of disease, with “syndrome” referring to a more than just localised inflammatory or mechanical obstruction of the sinonasal cavities by nasal polyps
- Syndrome implicitly refers to a severe and chronic condition, with/without comorbidities and challenges to achieve disease control
- Syndrome implicitly refers to the need for a multispeciality approach in a substantial portion of patients where multispeciality collaboration is warranted.

In case the ENT community would adopt the term Nasal Polyp Syndrome in the future, the patients of the PAB of EUFOREA are convinced that this might represent a step towards better recognition of the disease by the healthcare community and society as a whole.

## Conclusion

From the patient's perspective, the need for a lay language name for Chronic Rhinosinusitis with Nasal Polyps (CRSwNP) is evident. A lay name that acknowledges the multifaceted impact on patients can foster greater empathy and understanding from healthcare providers, family, and society. By adopting a more patient-centred and inclusive name, we can encourage a more compassionate approach to patient care and support, thereby strengthening the doctor-patient relationship and enhancing treatment adherence which is crucial to ensure better communication and patient understanding.

## Data Availability

The original contributions presented in the study are included in the article/Supplementary Material, further inquiries can be directed to the corresponding author.
